# The prognostic value of PKM2 and its correlation with tumour cell PD-L1 in lung adenocarcinoma

**DOI:** 10.1186/s12885-019-5519-2

**Published:** 2019-03-29

**Authors:** Chang-Ying Guo, Qian Zhu, Fang-Fang Tou, Xiao-Ming Wen, Yu-Kang Kuang, Hao Hu

**Affiliations:** 1 Department of Thoracic Surgery, Medical College of Nanchang University, Nanchang, 330006 China; 2 Department of Thoracic Surgery, Jiangxi Cancer Hospital, No. 519 Beijing East Road, Nanchang, 330006 China; 3 Department of Thoracic Surgery, Ji’an Central Hospital, Ji’an, 343000 China; 4 Department of Biotherapy, Sun Yat-sen University Cancer Center, Guangzhou, 510060 China

**Keywords:** Lung adenocarcinoma, Pyruvate kinase M2, Programmed death ligand 1, Prognostic factor

## Abstract

**Background:**

The prognostic value of PKM2 and its correlation with tumour cell PD-L1 in lung adenocarcinoma (LUAD) is unclear.

**Methods:**

A total of 506 lung adenocarcinoma samples from The Cancer Genome Atlas (TCGA) dataset and 173 LUAD tumour tissues from Jiangxi Cancer Hospital were used to analyse the correlation between PKM2 and PD-L1 expression. We further established a stable LUAD cell line with PKM2 knockdown and confirmed the association via Western blotting and flow cytometry analysis. Moreover, the prognostic values of PKM2 and PD-L1 were evaluated by the Kaplan-Meier method and Cox proportional hazards models.

**Results:**

Based on the above two large cohorts, we found that PKM2 was significantly positively associated with PD-L1 expression (r = 0.132, *P* = 0.003 and r = 0.287, *P* < 0.001, respectively). Subsequently, we found that PKM2 knockdown substantially inhibited PD-L1 expression in the A549 LUAD cell line. Moreover, survival analysis showed that higher expression of PKM2 was correlated with significantly shorter overall survival (OS) and disease-free survival (DFS) in lung adenocarcinoma patients (*P* < 0.001 and *P* = 0.050, respectively). Subgroup analysis showed that lung adenocarcinoma patients who expressed high PKM2 and PD-L1 levels experienced the poorest OS and DFS. Additionally, multivariate analysis suggested that high PKM2 and PD-L1 expression was an independent prognostic indicator for worse OS and DFS (HR = 1.462, *P* < 0.001 and HR = 1.436, *P* = 0.004, respectively).

**Conclusions:**

Our results demonstrated that PKM2 regulated PD-L1 expression and was associated with poor outcomes in lung adenocarcinoma patients.

## Background

Non-small-cell lung cancer (NSCLC) accounts for more than 85% of lung cancer cases, with approximately two-thirds of NSCLC patients presenting at an advanced stage [1]. Although molecular targeted treatment for NSCLC has improved clinical outcomes for patients with amenable mutations [2–4], only a fraction of patients have these mutations, and acquired resistance to targeted therapies frequently occurs [5]. Hence, the identification of new and efficient therapeutic targets for NSCLC remains an unmet need.

Pyruvate kinase isoform M2 (PKM2), a metabolic enzyme that catalyses the later steps of glycolysis, plays a key role in regulating metabolic activities in cancer cells and is necessary for tumour growth [6]. In addition to its known role as a metabolic enzyme, PKM2 can act as a signalling modulator in cancer development and progression [7, 8]. Previously, we identified that elevated expression of PKM2 is involved in cell proliferation and tumour formation in CD44^+^ A549 lung adenocarcinoma stem cells [9]. More importantly, we found that enhanced PKM2 expression contributes to stress resistance and therapeutic resistance in CD44^+^ A549 lung adenocarcinoma stem cells, suggesting PKM2 as a potential target for lung adenocarcinoma therapy [9]. Recently, Zhang et al. demonstrated that PKM2 interacts with intracellular suppressor of cytokine signalling 3 to decrease ATP production and impair the antigen-presenting abilities of dendritic cells in tumours [10]. Another study suggested that PKM2 regulates hypoxia-inducible factor 1α (HIF-1α) activity, interleukin-1β (IL-1β) induction and IL-10 production, thus promoting the inflammatory response [11]. Overall, these studies indicated that PKM2 could act as a modulatory effector on immune and inflammatory responses.

Interestingly, Eva M. Palsson-McDermott et al. found that PKM2 regulated immune checkpoint PD-L1 expression on tumour and immune cells in the CT26 colon carcinoma animal model [12]. However, the potential effects of PKM2 in regulating PD-L1 expression in lung adenocarcinoma remain unclear. Considering that the targeting of programmed death ligand 1 (PD-L1) has shown promise in patients with advanced NSCLC [13–15], we evaluated the association between PKM2 and PD-L1 in lung adenocarcinoma. Recently, Sun et al. evaluated the potential prognostic value of PKM2 in lung adenocarcinoma [16]. Nevertheless, there were several limitations in their study. First, the cohort was relatively small with only 65 patients, and approximately half of patients (47.6%) were diagnosed with stage IV disease. Moreover, the authors conducted univariate analysis of recurrence-free survival rather than multivariate analysis of overall survival, which prevented the data from supporting the conclusions drawn. Thus, the prognostic effect of PKM2 expression in lung adenocarcinoma patients remained unclear.

In the present study, which encompasses two large independent cohorts, we found a positive correlation between PKM2 and PD-L1 expression in lung adenocarcinoma patients. Subsequently, we found that the knockdown of PKM2 significantly reduced the total and surface protein levels of PD-L1. Finally, we evaluated the prognostic effect of these two proteins in lung adenocarcinoma. Overall, our findings identified a novel mechanism for the metabolic control of immune escape via the regulation of PD-L1 expression in lung adenocarcinoma. These results, along with previous findings regarding the role of PD-L1 in regulating tumour cell metabolism in tumours [17], provide a rationale for combining PKM2 targeting with PD-L1 inhibitors in the treatment of lung adenocarcinoma.

## Methods

### Patients and samples

This was a retrospective study with a cohort of 173 primary lung adenocarcinoma patients with histologically confirmed and surgically resected tumours at the Thoracic Surgery Department from January 2010–June 2014 at Jiangxi Cancer Hospital (Nanchang, China). Patients who had received neoadjuvant therapy before surgery, including chemotherapy, radiotherapy or immunotherapy, were excluded. All patients underwent lobectomy with additional radical lymph node dissection. Approval was obtained from the Institutional Ethical Board of Jiangxi Cancer Hospital. Verbal informed consent was provided by each patient before their tumour tissue samples were obtained. All procedures involving human samples were conducted according to the Declaration of Helsinki.

### Immunohistochemistry (IHC)

Slides (3 slides per sample) from formalin-fixed paraffin-embedded lung adenocarcinoma tissues were used for the histologic evaluation of PKM2 and PD-L1 expression by IHC. Briefly, 4-μm tissue sections were deparaffinized and subjected to heat-induced antigen retrieval. The slides were incubated overnight at 4 °C with a rabbit anti-human PKM2 mAb (1:100; D78A4, Cell Signaling Technology, Danvers, MA, USA) or rabbit anti-human PD-L1 mAb (1:100; E1L3N, Cell Signaling Technology). After incubation with horseradish peroxidase-conjugated secondary antibody (Envision™ Detection Kit, GK500705, Genetech), diaminobenzidine was used to visualize the staining.

### Scoring of PKM2 and PD-L1 expression

Staining for PKM2 was scored based on a 12-point semi-quantitative scoring system [18, 19]. PKM2 expression was quantified after evaluating both the intensity of the staining (0 = negative, 1 = weakly positive, 2 = moderately positive, and 3 = strongly positive) and the distribution of positively stained cells (0 = 0%, 1 = 1 to 10%, 2 = 11 to 50%, 3 = 51 to 80%, and 4 = 81 to 100%). The final histological score was determined as the staining intensity × distribution, with a range from 0 to 12. The cut-off point was the median (score < 8, negative expression and ≥ 8, positive expression).

The scores corresponding to PD-L1 expression on tumour cells (TC-PD-L1) were evaluated as a percentage of positively stained cells in the overall section with tumour cells as described in previous studies [20, 21]. Consistent with many other studies [22, 23], PD-L1 positivity was considered membranous PD-L1 expression in ≥5% of tumour cells. Two independent investigators assessed all slides without prior knowledge of the clinical outcome. When any discrepancies occurred between the two investigators, a consensus was reached by discussion.

### Cell line

The human lung adenocarcinoma A549 cell line was purchased from American Type Culture Collection. Cells were routinely maintained in RPMI-1640 basic medium supplemented with 10% FBS (Gibco, Life Technologies), 100 units/mL penicillin and 100 mg/mL streptomycin at 37 °C in a humidified atmosphere with 5% CO_2_.

### Establishment of stable PKM2 knockdown cells

Lentiviruses expressing PKM2-specific shRNAs (designated PKM2-shRNAs) or the negative control vector (designated scramble-shRNA) were purchased from GenePharma Inc. (Shanghai, China, http://www.genepharma.com). A549 cells were cultured at 1 × 10^6^ cells per well in 6-well plates and infected with PKM2-shRNA and scramble-shRNA lentiviruses in accordance with the manufacturer’s instructions. Target sequences with high PKM2 knockdown efficiency (PKM2-shRNA-1: 5′-CCGGCTACCACTTGCAATTATTTGACTCGAGTCAAATAATTGCAAG-TGGTAGTTTTTG-3′ and PKM2-shRNA-2: 5′-CCGGGCTGTGGCTCTAGACACTAAACTCGAGTTTAGTGTCTAGAGCCACAGCTTTTTG-3′) and the scrambled sequence without any effect on PKM2 levels (5′-CCGGGAGGCTTCTTATAAGTGTTTACTCGAGTAAACACTTATAAGAAGCCTCTTTTTG-3′) were adopted as described previously [6]. After 72 h, stable PKM2 knockdown cells were selected with puromycin (1 μg/mL).

### Western blotting

Cellular PKM2 and PD-L1 protein levels in lung adenocarcinoma cell lines were evaluated in total cell extracts by Western blot analysis. A total of 30 μg of protein was fractionated using SDS/PAGE and then transferred onto polyvinylidene fluoride membranes (Immobilon P; Millipore, Bedford, MA, USA). After blocking with 5% non-fat milk and washing with PBS, the membranes were incubated with rabbit anti-PKM2 mAb (1:1000; D78A4, Cell Signaling Technology, Inc., USA), rabbit anti-PD-L1 mAb (1:1000; E1L3N, Cell Signaling Technology, Inc., USA) and rabbit anti-Glyceraldehyde-3-phosphate dehydrogenase (GAPDH) mAb (1:5000, Cell Signaling Technology, Inc., USA), followed by the corresponding secondary antibodies (1:10000, sc-2004, Santa Cruz Biotechnology). The protein bands were visualized via Chemidoc Touch (Bio-Rad) and quantified relative to GAPDH expression using ImageJ software (NIH, Bethesda, MD, USA).

### RNA extraction and qRT-PCR

Total RNA was isolated from the cell lines using TRIzol reagent (Invitrogen, Carlsbad, CA, USA) and reverse transcribed using a PrimeScript RT™ Master Mix (Catalogue no: RR036A; Takara Biotechnology Co., Ltd.), followed by quantitative polymerase chain reaction (qPCR) with GoScript qPCR Master Mix (Promega; Madison, WI, USA) according to the manufacturer’s protocol.

The primers for PKM2 were forward 5′-ATTATTTGAGGAACTCCGCCGCCT-3′ and reverse 5′- ATTCCGGGTCACAGCAATGATGG -3′.

The primers for GAPDH were forward 5′-AAGGTCATCCCTGAGCTGAA-3′ and reverse 5′-TGACAAAGTGGTCGTTGAGG-3′.

GAPDH was used as an internal control, and the relative levels of mRNA were calculated by the 2[−∆∆CT] method.

### Flow cytometry

A total of 1 × 10^6^ cells were harvested and then stained with PE-conjugated anti-PD-L1 antibody (Cat# 557924, BD Biosciences) or with the corresponding isotype-matched controls (Cat# 555749, BD Biosciences) for 30 min at room temperature. Cells were run on a Gallios Flow Cytometer (Beckman Coulter) and analysed using FlowJo software (TreeStar).

### Analysis of TCGA data

Data regarding mRNA expression, mutational status and survival time from The Cancer Genome Atlas for Lung Adenocarcinoma (TCGA, Provisional) were downloaded from cBioPortal (http://cbioportal.org). RNA-Seq V2 RSEM was utilized to analyse mRNA expression. The cut-off for high/low PKM2 based on mRNA expression for survival analysis was determined by Cutoff Finder. For the *CD274* (*PD-L1*) gene, the cut-off for the PD-L1 high/low group was recorded according to the positive rate of PD-L1 expression by IHC and then checked for an association with survival. The Mann-Whitney test was used to compare PD-L1 levels between tumours with high PKM2 levels and tumours with low PKM2 levels, as PD-L1 levels were not normally distributed (*P* < 0.05 from the Kolmogorov-Smirnov normality test). Moreover, we compared survival differences between the high PKM2/high PD-L1 group and the low PKM2/low PD-L1 group.

### Statistical analysis

Statistical analyses were performed using SPSS 22.0 (IBM Corp., Armonk, NY, USA) and GraphPad Prism 6.07 (GraphPad Software, Inc., La Jolla, CA, USA). Continuous variables were presented as the mean ± standard deviation and compared by unpaired Student’s t tests. The chi-square test or Spearman’s rank correlation analysis was used for the analysis of PKM2 and PD-L1 expression levels in tumour cells as categorical or continuous variables, respectively. The log-rank test was applied to test the relationship between mRNA expression and overall survival (OS)/disease-free survival (DFS), and the Kaplan-Meier method was used to depict survival curves. Cox proportional hazards regression models were used to perform univariate and multivariate survival analyses, and only variables whose *P*-values were less than 0.1 in the univariate analysis were included in the multivariate analysis. All statistical significance was set at *P* < 0.05 (two-sided).

## Results

### Patient clinicopathological features

**Fig. 1:**
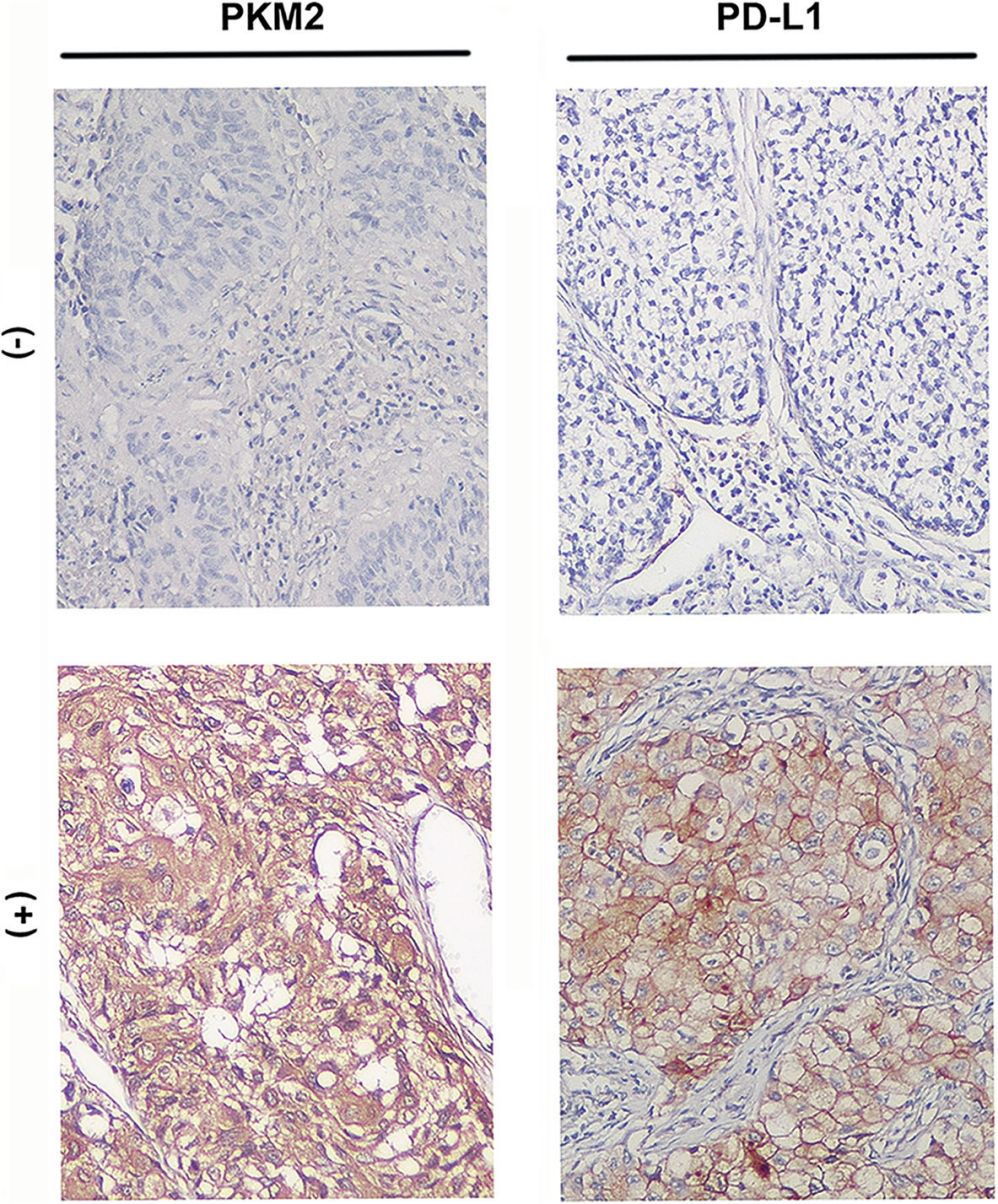
Representative images of PKM2 and PD-L1 expression in lung adenocarcinoma tumour cells. (× 400 magnification)

**Fig. 2:**
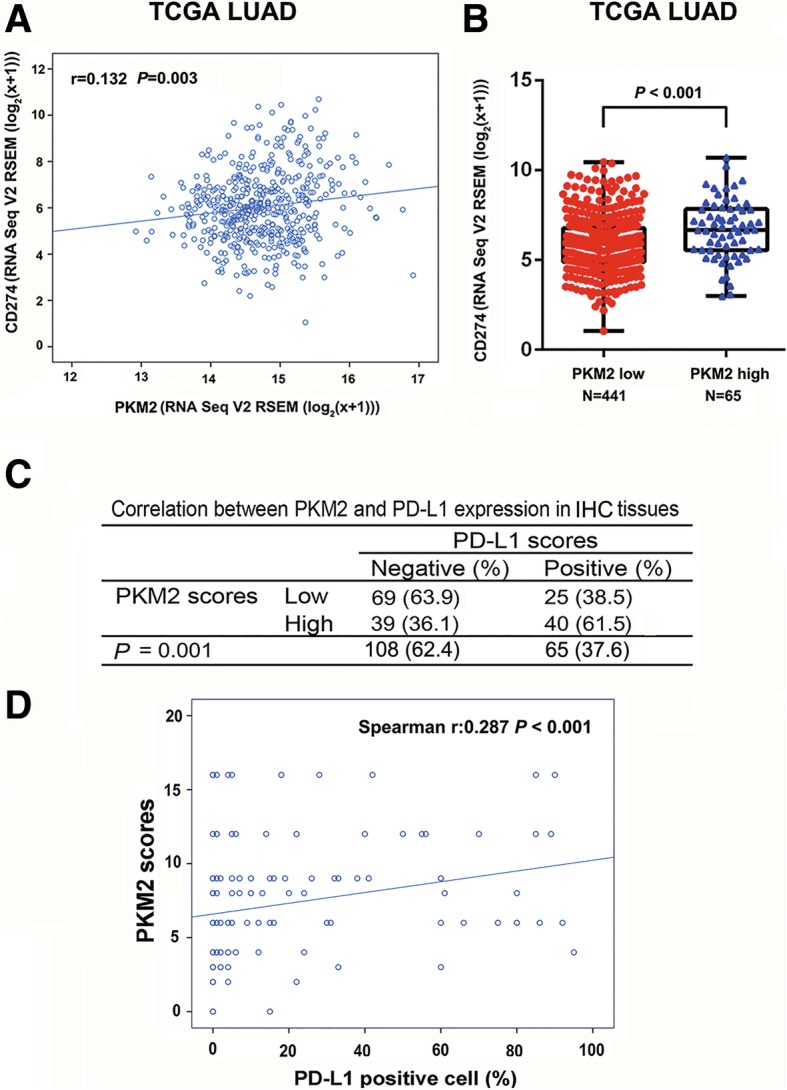
Correlation of PKM2 with PD-L1 in lung adenocarcinoma. (**a**) Correlation between PKM2 and PD-L1 mRNA expression in The Cancer Genome Atlas (TCGA) lung adenocarcinoma (LUAD) cohort (*n* = 506) (*P* = 0.003). (**b**) PD-L1 expression in patients with high PKM2 (*n* = 441) and low PKM2 (*n* = 65) expression (*P* < 0.001). (**c**) The chi-square test was employed to assess the correlation between PKM2 and PD-L1 expression in samples as categorical variables. (**d**) Spearman’s rank correlation analysis was used to evaluate the strength of the association between PKM2 scores and PD-L1 expression in the tumour cells as continuous variables. ^*^P < 0.05, ^**^P < 0.01. A box-and-whisker plot was used to represent the data. The box plot represents the first (lower bound), median and third (upper bound) quartiles

The baseline clinicopathological characteristics of 173 primary lung adenocarcinoma tissue samples by IHC are listed in Table 1. The average age was 47 y (range 27–67 y). There were 124 (71.7%) female patients, and the majority of patients were never smokers (68.2%). Fifty-one (29.5%) patients were diagnosed with stage I, 66 (38.2%) with stage II, 43 (24.9%) with stage III, and 13 (7.5%) with stage IV disease. For the major driver mutational status, mutations in epidermal growth factor receptor (*EGFR*) and Kirsten rat sarcoma viral oncogene homologue (*KRAS*) were detected in 20 (11.6%) and 25 (14.4%) patients, respectively. Anaplastic lymphoma kinase (*ALK*) translocations were found in eight patients (4.6%) and were mutually exclusive with mutations in *EGFR* and *KRAS*. Immunohistochemical analysis of lung adenocarcinoma tumour tissues showed that PKM2 was primarily expressed in the cytoplasm of tumour cells, whereas PD-L1 was expressed both on the membrane and in the cytoplasm of tumour cells (Fig. 1). Considering that PD-L1 is mainly expressed on the cell surface, we only evaluated the membrane expression of PD-L1 and termed it TC-PD-L1. Finally, 65 (37.6%) cases displayed positive TC-PD-L1 staining (Fig. 2C). We next explored the correlation between PKM2 expression and tumour biology and observed that PKM2 expression was associated with N classification (*P* = 0.006) and TNM stage (*P* = 0.005); however, no significant correlation was observed between PKM2 expression and T classification (*P* = 0.114), M classification (*P* = 0.232) (Table 1). Nevertheless, no strong relationships were detected between PKM2 expression and other clinicopathological characteristics, including patient age, sex and smoking history.

**Table 1:** Association between *PKM2* expression and clinicopathological features of 173 LUAD patients

Characteristic	No. of patients (%)	*PKM2* expression		*P*-value
		Low	High	
Age (years), median (range)	47 (27–67)			
≤ 60	149 (86.1%)	77 (51.7%)	72 (48.3%)	0.080
> 60	24 (13.9%)	17 (70.8%)	7 (29.2%)	
Sex				
Male	124 (71.7%)	67 (54.0%)	57 (46.0%)	0.899
Female	49 (28.3%)	27 (55.1%)	22 (44.9%)	
Smoking history				
Never	118 (68.2%)	64 (54.2%)	54 (45.8%)	0.970
Current/Former	55 (31.8%)	30 (54.5%)	25 (45.5%)	
pT classification				
T1	48 (27.7%)	33 (68.8%)	15 (31.3%)	0.114
T2	67 (38.7%)	31 (46.3%)	36 (53.7%)	
T3	46 (26.6%)	24 (52.2%)	22 (47.8%)	
T4	12 (6.9%)	6 (50.0%)	6 (50.0%)	
pN classification				
N0	100 (57.8%)	62 (62.0%)	38 (38.0%)	**0.006**
N1	47 (27.2%)	25 (53.2%)	22 (46.8%)	
N2–3	26 (15.0%)	7 (26.9%)	19 (73.1%)	
pM classification				
M0	160 (92.5%)	89 (55.6%)	71 (44.4%)	0.232
M1	13 (7.5%)	5 (38.5%)	8 (61.5%)	
pTNM stage				
I	51 (29.5%)	38 (74.5%)	13 (25.5%)	**0.005**
II	66 (38.2%)	33 (50.0%)	33 (50.0%)	
III	43 (24.9%)	18 (41.9%)	25 (58.1%)	
IV	13 (7.5%)	5 (38.5%)	8 (61.5%)	
Positive *EGFR* mutational status	20 (11.6%)	12 (12.8%)	8 (10.1%)	
Positive *KRAS* mutational status	25 (14.4%)	14 (14.9%)	11 (13.9%)	
Positive *ALK* translocation status	8 (4.6%)	5 (5.3%)	3 (3.8%)	

*PKM2*, pyruvate kinase M2; *EGFR*, epidermal growth factor receptor; *KRAS*, Kirsten rat sarcoma viral oncogene homologue; *ALK*, anaplastic lymphoma kinase; LUAD, lung adenocarcinoma

### Correlation between PKM2 and PD-L1 in lung adenocarcinoma patients

First, we evaluated the correlation between PKM2 and PD-L1 based on mRNA expression using TCGA lung adenocarcinoma (LUAD, 506 samples) dataset. A positive correlation between PKM2 and PD-L1 was detected in TCGA LUAD cohort (r = 0.132, *P* = 0.003) (Fig. 2A). Subsequently, we compared PD-L1 expression between the PKM2 low (*n* = 441) and high (*n* = 65) expression groups and found that PD-L1 levels were significantly lower in patients with low PKM2 levels than in patients with high PKM2 levels (*P* < 0.001) (Fig. 2B). Moreover, we evaluated the correlation between PKM2 and PD-L1 protein expression in 173 primary lung adenocarcinoma tissue samples by IHC. We found that the protein expression of PKM2 was significantly correlated with TC-PD-L1 expression, assessed as categorical variables (Fig. 2C, *P* = 0.001). A similar positive association was observed when PKM2 and PD-L1 levels were assessed as continuous variables. Spearman’s rank correlation analysis suggested a positive correlation between PKM2 and PD-L1 expression levels (r = 0.287, *P* < 0.001; Fig. 2D). Therefore, there is a positive correlation between PKM2 and PD-L1 expression in lung adenocarcinoma patients.

### PKM2 regulates PD-L1 expression in the A549 lung adenocarcinoma cell line

**Fig. 3:**
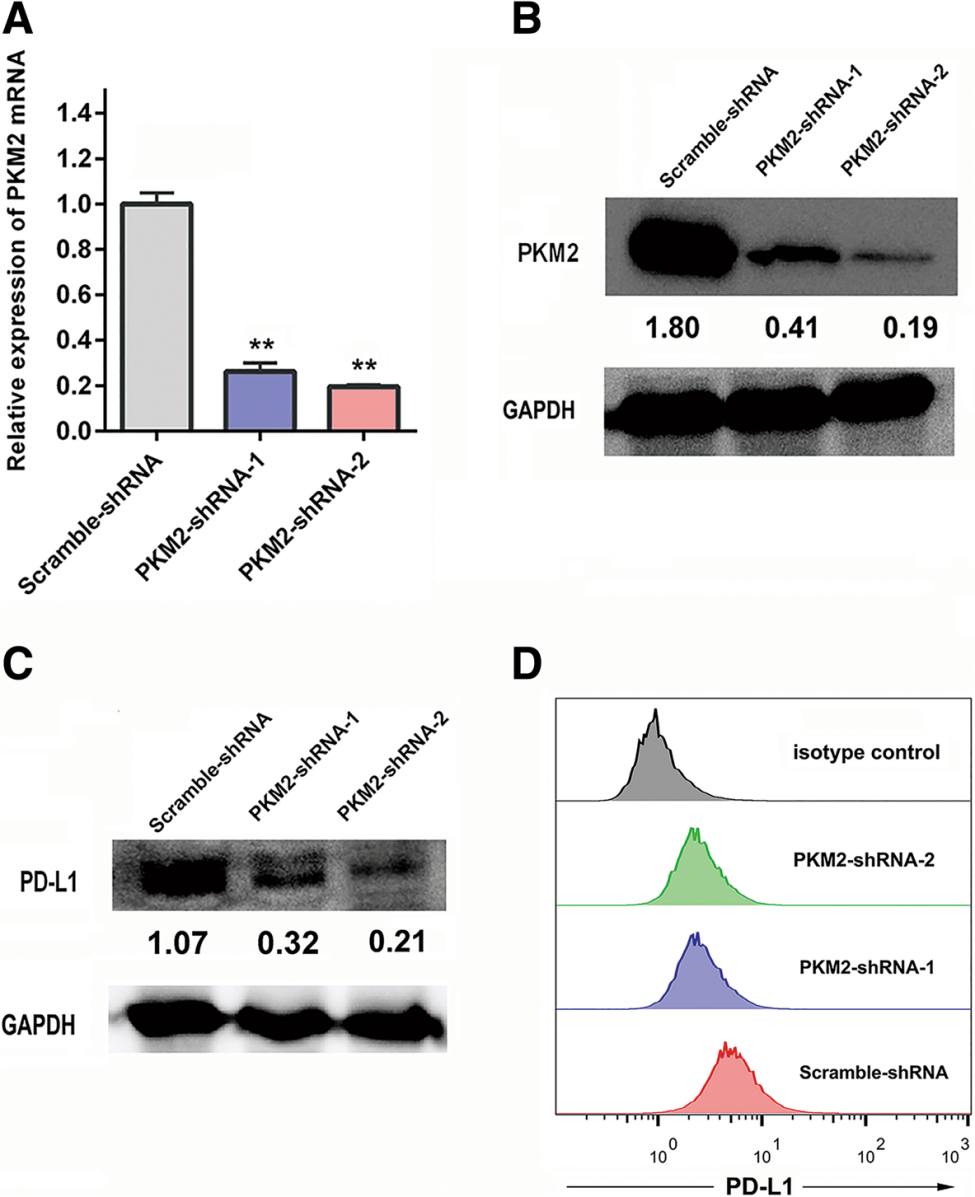
Regulation of PD-L1 expression by PKM2 in a lung adenocarcinoma cell line. PKM2 expression in A549 lung adenocarcinoma cells transfected with PKM2-shRNA and scramble-shRNA lentiviruses. Seventy-two hours later, the cells were subjected to puromycin selection (1 μg/mL) for 2 weeks. Subsequently, the cells were collected for qRT-PCR and protein analysis. Quantification of both qRT-PCR and Western blotting data showing that the knockdown of PKM2 with shRNA-1 and shRNA-2 markedly suppressed PKM2 mRNA levels (**a**) and total protein expression (**b**) compared with those with scrambled control. (**c**) Western blotting of total PD-L1 protein levels in PKM2-shRNA-1-, PKM2-shRNA-2- and scramble-shRNA-transfected lung adenocarcinoma cell lines. (**d**) Flow cytometric analysis of cell surface PD-L1 levels in PKM2-shRNA-1-, PKM2-shRNA-2- and scramble-shRNA-transfected lung adenocarcinoma cell lines. PKM2-shRNA1, blue zone; PKM2-shRNA2, green zone; scramble-shRNA, red zone; isotype control, grey zone. Unpaired t tests were used to calculate the two-sided *P*-values. ^*^P < 0.05, ^**^P < 0.01. Error bars in the bar charts represent standard deviation

To further understand the relationship between PKM2 and PD-L1 in lung adenocarcinoma tumour cells, we generated stable PKM2 knockdown cells by transfection with PKM2-specific short hairpin RNAs (shRNAs). The levels of PKM2 expression in stable PKM2 knockdown cells were confirmed by both qRT-PCR and Western blotting (Fig. 3A-B). We detected PD-L1 protein expression in stable PKM2 knockdown cells via Western blotting analysis and found that the protein level of PD-L1 was substantially downregulated (Fig. 3C). Moreover, we investigated cell surface PD-L1 protein levels (by flow cytometry) in the PKM2-shRNA and scramble-shRNA groups. The results of flow cytometry analysis revealed that the expression level of PD-L1 was more dramatically reduced in the PKM2-shRNA group compared to that in the scramble-shRNA group (Fig. 3D).

### Prognostic analysis of PKM2 and PD-L1 expression in lung adenocarcinoma

**Fig. 4:**
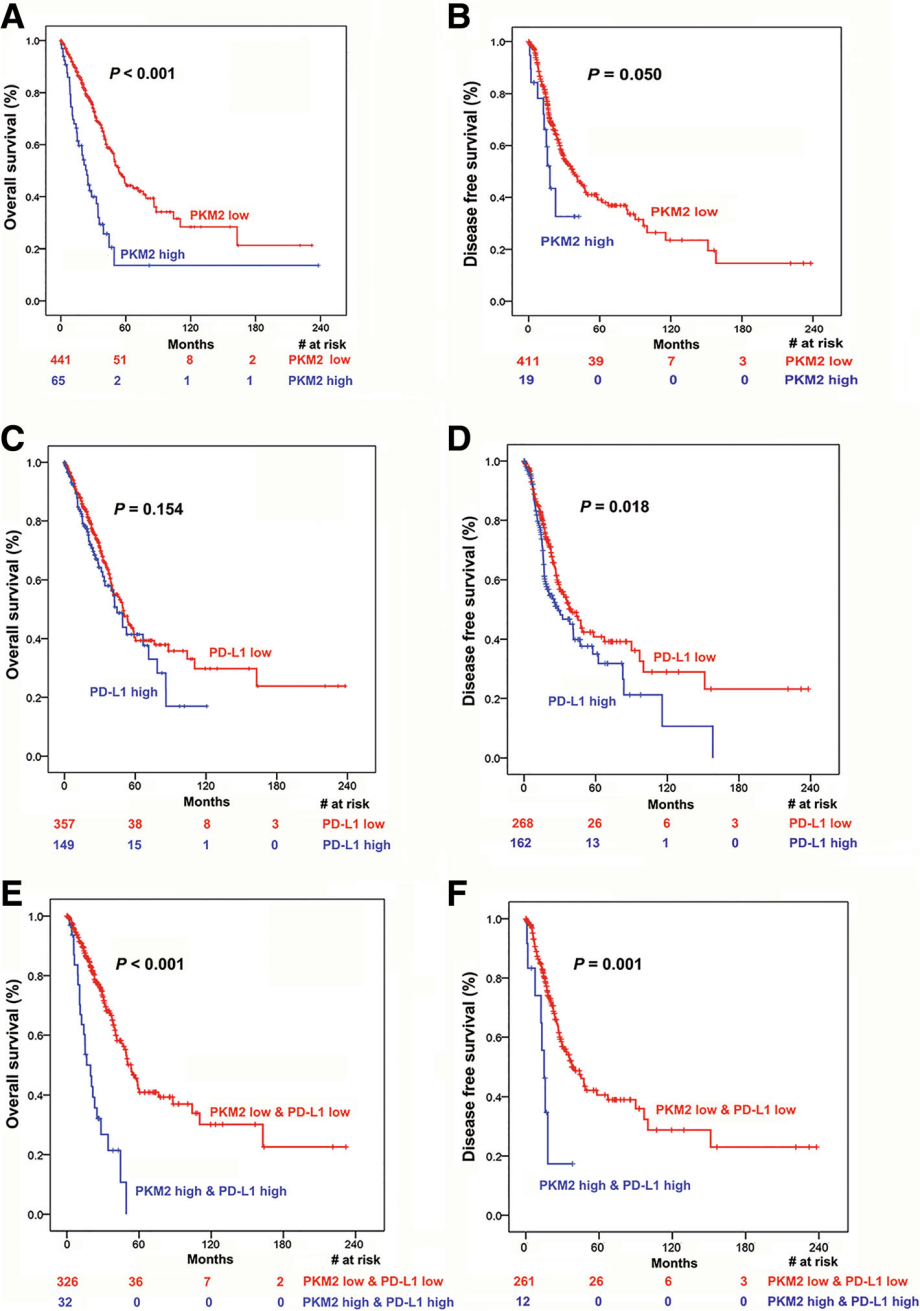
Prognostic analysis of PKM2 and PD-L1 expression in lung adenocarcinoma. (**a**-D) Kaplan-Meier analysis of overall survival and disease-free survival according to PKM2 expression (**a** and **b**) and PD-L1 (**c** and **d**) expression in TCGA LUAD patient cohorts. (**e** and **f**) Kaplan-Meier analysis of overall survival and disease-free survival according to the combination of PKM2 and PD-L1 expression in TCGA LUAD patient cohorts. Patients with high PKM2 and high PD-L1 levels had the poorest overall survival (**e**) and disease-free survival (**f**) than patients with low PKM2 and low PD-L1 levels or patients with low PKM2 levels alone (**a** and **b**). The log-rank test was used to determine the association between mRNA expression and overall survival/disease-free survival, and the Kaplan-Meier method was used to generate survival curves. *LUAD* lung adenocarcinoma; *TCGA* The Cancer Genome Atlas; Log-rank test, two-sided. The number of patients at risk among these groups at different time points is presented at the bottom of the graphs

**Fig. 5:**
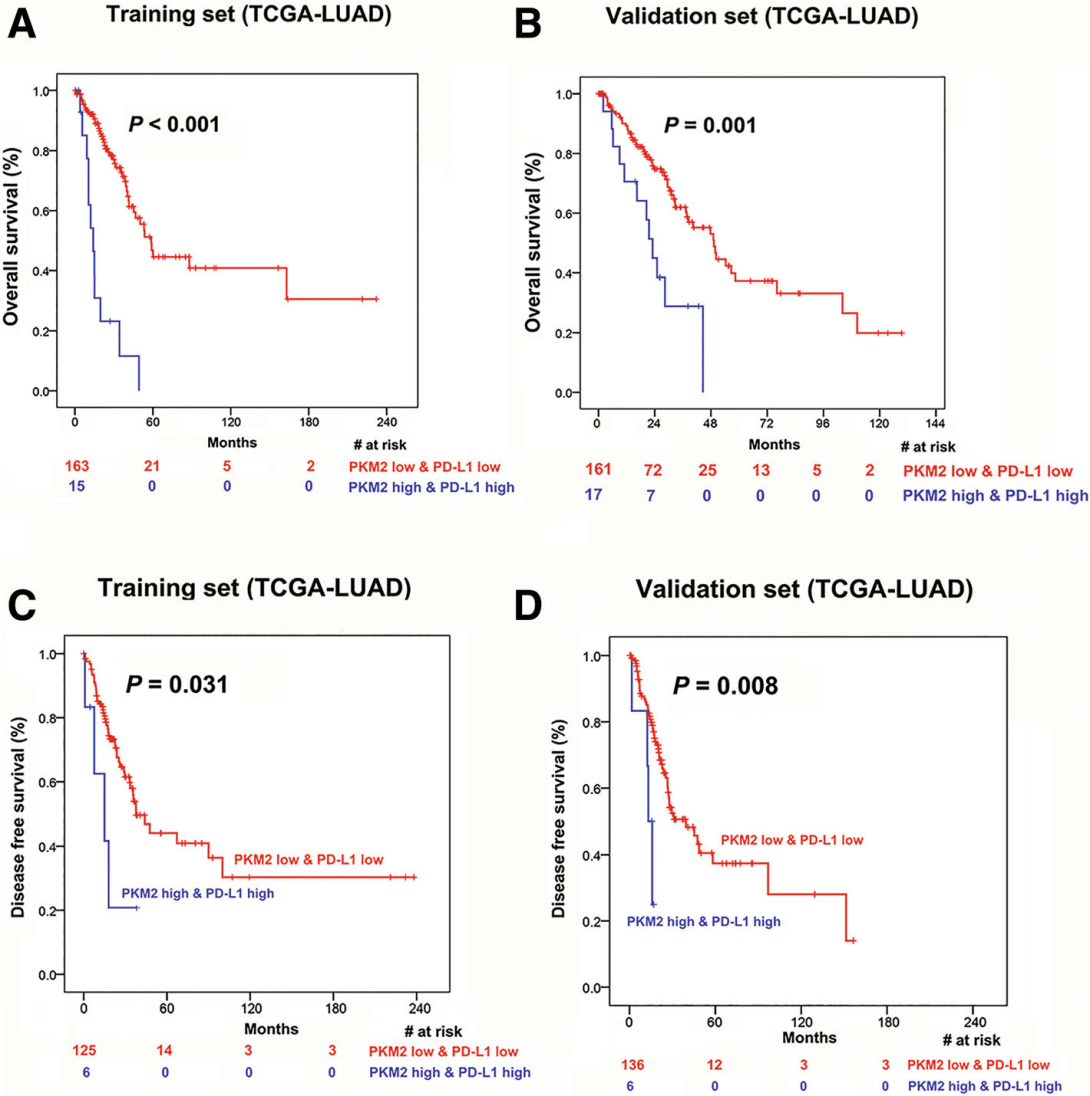
Association of the combination of PKM2 and PD-L1 expression with prognosis in patients with lung adenocarcinoma in the training and validation cohorts. Kaplan-Meier curves for overall survival and disease-free survival according to the combination of PKM2 and PD-L1 expression in the training (**a** and **b**) and validation (**c** and **d**) lung adenocarcinoma patient cohort from TCGA (TCGA LUAD). The number of patients at risk in the high PKM2 & high PD-L1 and low PKM2 & low PD-L1 groups at different time points are presented at the bottom of the graphs

Kaplan-Meier survival analysis was performed in TCGA LUAD cohort to compare OS and DFS according to PKM2 and PD-L1 expression. Among the 605 patients included in the survival analysis, positive *EGFR* mutations, *KRAS* mutations and *ALK* translocations were detected in 34 (6.71%), 74 (14.6%) and 32 (6.32%) patients, respectively. To explore the possible relationships between survival and PKM2 and PD-L1 levels, we divided TCGA LUAD cohort into percentiles based on mRNA expression and identified cut-off points for low and high PKM2 at 0.871 and 0.955 for OS and DFS analyses, respectively. Patients with high PKM2 expression had a significantly shorter OS and DFS than patients with low PKM2 expression (*P* < 0.001 and *P* = 0.050, respectively; Fig. 4A-B). Moreover, the cut-off point was 0.624 and 0.628 for low and high PD-L1 expression for OS and DFS analyses, respectively. We found no statistically significant difference in OS among patients with high vs. low PD-L1 (*P* = 0.154), although patients with high PD-L1 expression seemed to have poorer OS than patients with low PD-L1 expression (Fig. 4C). However, higher PD-L1 expression remained associated with shorter DFS (*P* = 0.018, Fig. 4D). Next, we tested whether the combined expression of PKM2 and PD-L1 could improve survival differences between groups (high PKM2 & high PD-L1 vs. low PKM2 & low PD-L1) (Fig. 4E-F). Interestingly, we observed that the separation based on OS and DFS between these two groups (*P* < 0.001 for both) was more apparent than the separation between groups according to PKM2 expression alone (Fig. 4A-B, respectively). To make our results more conclusive, we used random number generators to randomly assign the available set of samples into two groups (a training set and a validation set) in a 1:1 ratio and validated the findings in the two groups (Fig. 5). Finally, we performed univariate and multivariate Cox regression analyses for OS and DFS. All significant clinical factors (*P*-values less than 0.1) in the univariate analysis were included in the multivariate Cox regression analysis (Table 2). As shown in Table 2, high expression of PKM2 and PD-L1 still powerfully and independently predicted poor OS and DFS, indicating that the concomitant PKM2 and PD-L1 expression could be a useful biomarker of response to therapy.

**Table 2:** Univariate and multivariate Cox regression analyses for OS and DFS in LUAD cohorts

Variables	OS		DFS	
	HR (95% CI)	*P*-value	HR (95% CI)	*P*-value
	Univariate analysis			
Age (> 65 vs. ≤65)	1.390 (0.985–1.963)	0.061	1.242 (0.852–1.811)	0.260
Sex (female vs. male)	1.019 (0.723–1.437)	0.914	1.119 (0.770–1.627)	0.555
pT (pT3–4 vs. T1–2 + Tx)	2.391 (1.487–3.843)	< 0.001	2.629 (1.536–4.501)	< 0.001
pN (pN+ vs. N0 + Nx)	2.698 (1.908–3.814)	< 0.001	2.236 (1.524–3.280)	< 0.001
pM (pM1 vs. M0 + Mx)	2.049 (1.128–3.723)	0.018	2.005 (0.877–4.584)	0.099
PKM2 high & PD-L1 high vs. PKM2 low & PD-L1 low	1.581 (1.359–1.840)	< 0.001	1.481 (1.162–1.889)	0.002
	Multivariate analysis			
Age (> 65 vs. ≤65)	1.639 (1.142–2.353)	0.007		
pT (pT3–4 vs. T1–2 + Tx)	1.740 (1.057–2.862)	0.029	2.178 (1.252–3.788)	0.006
pN (pN+ vs. N0 + Nx)	2.615 (1.823–3.753)	< 0.001	2.153 (1.461–3.174)	< 0.001
pM (pM1 vs. M0 + Mx)	1.188 (0.633–2.230)	0.592	1.658 (0.722–3.810)	0.233
PKM2 high & PD-L1 high vs. PKM2 low & PD-L1 low	1.462 (1.246–1.715)	< 0.001	1.436 (1.119–1.843)	0.004

Abbreviations: *HR* hazard ratio, *OS* overall survival, *DFS* disease-free survival, *CI* confidence interval, *LUAD* lung adenocarcinoma

## Discussion

NSCLC is the leading cause of cancer mortality worldwide, and its treatment options have encountered tremendous difficulties [24]. Several studies have revealed an oncogenic role of PKM2 in tumourigenesis and suggested that PKM2 could be a potential target for treating lung adenocarcinoma [6, 16, 25, 26]. Interestingly, recent studies reported that PKM2 was involved in the activation of various immune cells, including macrophages [11], dendritic cells [27], monocytes [28] and T cells [29]; however, the possible role of PKM2 in the regulation of the immune checkpoint PD-L1 in lung adenocarcinoma is unknown. In the present study, we demonstrate that PKM2 regulates TC-PD-L1 expression in lung adenocarcinoma using TCGA dataset, tissue samples and cell lines. Considering the importance of PD-L1 expression in NSCLC supported by the clinical benefit of anti-PD-L1 antibodies as single agents in NSCLC patients, these findings further support the investigation of PKM2 as a potential target in lung adenocarcinoma. Moreover, we found that lung adenocarcinoma patients with high PKM2 and PD-L1 expression had the poorest OS and DFS compared with those in patients with low expression of either factor or both factors, indicating that the concomitant PKM2 and PD-L1 expression provides important prognostic information for lung adenocarcinoma patients. This will be useful for the better selection and management of lung adenocarcinoma patients who will benefit from anticancer therapy.

Recent observations have suggested that PKM2 has a dual role in the regulation of immune cell function and antitumour immune responses. On the one hand, PKM2 is imperative for immune cell function and inflammatory responses. For example, PKM2 interacts with HIF-1α and activates the HIF-1α-dependent transcription of enzymes necessary for aerobic glycolysis, regulating lactate production and high-mobility group box 1 (HMGB1) release in macrophages [30]. Similarly, PKM2 is required for phagocytic activity, the expression of co-stimulatory molecules CD86 and CD80, and the activation of CD8^+^ T cells by myeloid dendritic cells [27]. On the other hand, PKM2 is involved in the generation of the tumour immune microenvironment. PKM2 enables the metabolic shift from oxidative phosphorylation to aerobic glycolysis and results in increased lactate production, leading to tumour immune evasion in the tumour microenvironment [31, 32]. Other evidence also suggests that cellular metabolism has an integral role in both tumour growth and antitumour immune responses in the tumour immune microenvironment [17, 33]. Overall, these data suggest that PKM2 might contribute to immune regulation in the tumour microenvironment. However, whether PKM2 contributes to tumour immune escape in lung adenocarcinoma is unknown. Here, we show a positive correlation between PKM2 and PD-L1 expression levels in lung adenocarcinoma samples. In addition, knockdown of PKM2 using shRNAs markedly reduced the total protein and cell surface protein levels of PD-L1 in lung adenocarcinoma cells. These findings demonstrate that the regulatory role of PKM2 in human lung adenocarcinoma PD-L1 expression. Our results are broadly consistent with recent findings that PKM2 upregulation contributes to PD-L1 expression in immune cells and CT-26 colon carcinoma cells [12, 34]. Collectively, our findings demonstrate that PKM2 is involved in the regulation of PD-L1 expression in human lung adenocarcinoma cells, indicating the potential of this enzyme as a target in the treatment of lung adenocarcinoma. Moreover, our results complement recent findings involving the role of PKM2 in immune regulation.

A previous study suggested that lung adenocarcinoma patients with higher PKM2 protein expression levels had a significantly shorter recurrence-free survival [16]. We demonstrated that higher PKM2 mRNA levels were associated with shorter OS and DFS in LUAD, further supporting the role of PKM2 expression as a negative prognostic marker in lung adenocarcinoma patients. Previous studies [35–38] have evaluated the prognostic effect of PD-L1 expression in NSCLC, but the results appeared conflicting and inconsistent. In our study, NSCLC patients with higher PD-L1 expression had worse DFS but not OS. Given the substantially greater prognostic value of multiple biomarkers than of a single biomarker [39], we next investigated whether the combination of PKM2 and PD-L1 expression could improve the prognostic value in lung adenocarcinoma and showed that patients with high PKM2 & high PD-L1 levels had shorter OS and DFS than patients with low PKM2 & low PD-L1 levels or patients with high PKM2 or PD-L1 levels alone. Moreover, multivariate Cox regression analyses showed that high expression of PKM2 and PD-L1 was an independent predictor of poor OS and DFS. These findings have important potential clinical applications for identifying patients who will benefit from therapy. However, further study is required to confirm this hypothesis before PKM2 and PD-L1 expression can be used as biomarkers in future clinical applications. Thus far, we have revealed that the combination of PKM2 and PD-L1 is a more powerful prognostic factor in patients with lung adenocarcinoma.

Although this is the first study evaluating the relationship between PKM2 and TC-PD-L1 expression in lung adenocarcinoma, several limitations of our study need to be noted. This study was retrospective in nature, and the sample size was relatively small, which could have biased the results. Hence, a larger independent and prospective study is needed to validate these results in the future. Moreover, other studies are necessary to determine the signalling pathways and factors involved in the regulation of TC-PD-L1 by PKM2 and to distinguish the effects of PKM2 on the tumour immune evasion pathway.

## Conclusions

In conclusion, we demonstrated that PKM2 contributes to TC-PD-L1 expression in lung adenocarcinoma. Moreover, the combination of PKM2 and PD-L1 expression may be a more useful prognostic factor for identifying lung adenocarcinoma patients who might benefit from therapy.

## Abbreviations

ALK: anaplastic lymphoma kinase
DFS: disease-free survival
EGFR: epidermal growth factor receptor
HIF-1α: hypoxia-inducible factor 1α
HMGB1: high-mobility group box 1
IHC: immunohistochemistry
IL-1β: interleukin-1β
KRAS: Kirsten rat sarcoma viral oncogene homologue
LUAD: lung adenocarcinoma
NSCLC: non-small-cell lung cancer
OS: overall survival
PD-L1: programmed death ligand 1
PKM2: pyruvate kinase isoform M2
qPCR: quantitative polymerase chain reaction
TCGA: The Cancer Genome Atlas
TCs: tumour cells
